# Editorial: Bacteriophages, prophages, and their products: regulating bacterial populations

**DOI:** 10.3389/fmicb.2025.1628912

**Published:** 2025-06-03

**Authors:** Barbara Maciejewska, Agata Dorotkiewicz-Jach, Zuzanna Drulis-Kawa, Gamaliel López-Leal

**Affiliations:** ^1^Department of Pathogen Biology and Immunology, University of Wroclaw, Wroclaw, Poland; ^2^Laboratorio de Biología Computacional y Virómica Integrativa, Centro de Investigación en Dinámica Celular, Universidad Autónoma del Estado de Morelos, Cuernavaca, Morelos, Mexico

**Keywords:** phages, phage therapy, phage diversity, bacteriophages, prophages, phage host interactions, phage evolution, antimicrobial resistance

Bacteriophages, the most numerous biological entities on Earth, can be found as separate particles in the environment as well as in various forms within bacterial genomes, ranging from intact inducible prophages to incomplete phage genes. As the main bacterial predator, they are powerful regulators of microbial populations and influence bacterial evolution by selection, and gene exchange. Moreover, both bacteriophages and their derivatives offer a critical solution to the growing threat of antimicrobial resistance. This Research Topic includes articles that contribute to broadening our knowledge in three themes: phage biology, ecology and evolution, and phage therapy and biocontrol. Together, they illustrate the multifaceted roles phages and their products play in bacterial evolution, community dynamics, and the development of novel antimicrobial strategies.

Several articles in this Research Topic expand our arsenal against resistant pathogens through the characterization of new bacteriophages and highlighting their therapeutic potential against multidrug-resistant pathogens and bacterial diseases of agricultural. Khazani Asforooshani et al. introduced a newly isolated *Enterococcus faecium* phage EF-M80 that shows a broad host range (against resistant strains), biofilm-degrading ability, and resilience to high temperatures and pH. Based on this, researchers developed a hydrogel-encapsulated version, which was applied in a wound infection mouse model and led to enhanced healing by increasing the presence of neutrophils, fibroblasts, blood vessels, and collagen. The results suggest that the hydrogel-encapsulated EF-M80 phage holds promising potential for the treatment of infections caused by antibiotic-resistant *E. faecium*. Shamsuzzaman et al. isolated and characterized four lytic phages from hospital sewage water (which belong to the *Tequatrovirus* and *Kuravirus* genus) with broad host ranges and demonstrated their efficacy in reducing *Escherichia coli* ST131 infections *in vivo*. This pandemic clone poses a significant challenge to current treatments, and their findings mark a critical step toward phage-based interventions. On the other hand, Köhne et al. isolated and characterized phages active against *Streptococcus equi* subsp. *Zooepidemicus* (a major causative agent of infections in foals and adult horses), revealing strong *in vitro* lytic activity and providing a basis for future development of phage therapeutics in veterinary medicine, and expanding the catalog of phages with therapeutic potential.

In agriculture, Chantapakul et al. tested phage cocktails—mostly of the families *Autographiviridae* and *Pootjesviridae*—against *Rhizobium radiobacter*, the causative agent of crown gall disease in blueberries. The study demonstrated a significant bacterial reduction in a soil-based model, pointing to promising phage applications in plant protection. Although temperate, Miller et al. reported a phage with a narrow host range that infects *Klebsiella quasipneumoniae* and exhibits features such as biofilm disruption and phage–antibiotic synergy. While temperate phages are not commonly used in phage therapy, characterizing these phage populations remains important, as they may hold therapeutic potential through genetic engineering.

Beyond their role as lytic agents, phages encode proteins with potent antimicrobial activity. In this sense, Tian et al. report a novel phage, PA1, showing broad lytic activity against *Pantoea ananatis*. Based on the phage PA1 genome, they identified and produced an endolysin (PA1-Lys) and a hypothetical protein (PA1-LRP), enhancing the activity of the endolysin PA1-Lys. These findings reveal new insight into phage-induced lysis mechanisms and indicate PA1-LRP as a novel lysis-related protein with potential applications in phage therapy. Moreover, Zhao et al. identified the 73-amino acid Icd protein from phage P1 as a potent inhibitor of *E. coli* growth. Icd directly binds the essential division protein FtsZ, disrupting Z-ring formation. What's more, a core region (aa 12–51) retains full antibacterial activity, highlighting a novel mechanism to suppress bacterial growth and a minimal peptide with potential for antibacterial design. Both articles help to better understand the phage-host relationship and provide clues for the development of new antimicrobial proteins.

In addition to the emphasis on the use of phages and phage enzymes in combating bacteria, it is equally important to advance our understanding of phage interactions with bacterial hosts, the dynamic nature of these relationships, and the broader ecological roles and diversity of phages. Barrio-Pujante et al. focus on minimizing phage resistance in *Klebsiella pneumoniae* by inhibiting Quorum Sensing (QS) with cinnamaldehyde (CAD). Results show that CAD effectively reduces QS activity, allowing phages to infect previously resistant *K. pneumoniae* strains. This combined treatment enhances phage proliferation and significantly reduces bacterial growth, suggesting QS inhibitors could be valuable in phage therapy and phage-antibiotic combinations to combat antimicrobial resistance. DiSalvo et al. explore the dynamics of bacteriophage interactions within the *Paraburkholderia-Dictyostelium discoideum* symbiosis system. This study is noteworthy due to the versatility of this genus and the interactions of different species with various hosts. By isolating and characterizing six environmental *Paraburkholderia* phages, the research examines their impact on bacterial symbionts and host amoebae. The study reveals diverse phage effects on symbiont infection prevalence and host fitness, highlighting the complexity of tripartite interactions and laying the groundwork for future investigations into phage therapy and bacterial evolution.

To use bacteriophages as alternative strategies to antibiotics, it is essential to identify and characterize prophage populations within key pathogens such as *Acinetobacter baumannii*. In this context, Arellano-Maciel et al. analyzed the prophage diversity in *A. baumannii* isolates from various geographic regions, identifying 13 major clusters and revealing the global prevalence of *Vieuvirus*-related prophages. Studies like this provide valuable insights into the diversity and distribution of prophages across regions. The identification of distinct geographically prophage populations underscores the importance of further research into how phage–host interactions evolve in different environments. On the other hand, Zaychikova et al. reported the isolation of Vic9, the first subcluster B2 mycobacteriophage identified in Russia. Although it lysed *Mycobacterium tuberculosis* with low efficiency, its genome revealed unique features—including a recombination event and queuosine biosynthesis genes—shedding light on phage evolution and host adaptation.

Finally, in this Research Topic, Hellwig et al. introduced a novel methodology using biorthogonal non-canonical amino acid tagging (BONCAT) and click chemistry to label and isolate phage-host complexes without cultivation. This approach paves the way for studying phages in complex microbial communities, enabling high-resolution detection of active infection events *in situ*.

